# EnhancedMulti-Scenario Pig Behavior Recognition Based on YOLOv8n

**DOI:** 10.3390/ani15192927

**Published:** 2025-10-09

**Authors:** Panqi Pu, Junge Wang, Geqi Yan, Hongchao Jiao, Hao Li, Hai Lin

**Affiliations:** 1 Key Laboratory of Efficient Utilization of Non-Grain Feed Resources (Co-Construction by Ministry and Province), Ministry of Agriculture and Rural Affairs, Shandong Provincial Key Laboratory of Animal Nutrition and Efficient Feeding, Department of Animal Science, Shandong Agricultural University, Tai’an 271017, China; 2 College of Water Resources and Civil Engineering, China Agricultural University, Beijing 100193, China

**Keywords:** pig, behavior recognition, precision livestock farming, YOLO, multi-scene detection

## Abstract

Advances in smart animal husbandry necessitate efficient pig behavior monitoring, yet traditional approaches suffer from operational inefficiency and animal stress. We address these limitations through a lightweight YOLOv8n architecture enhanced with SPD-Conv for feature preservation during downsampling, LSKBlock attention for contextual feature fusion, and a dedicated small-target detection head. Experimental validation demonstrates superior performance: the optimized model achieves a 92.4% mean average precision (mAP@0.5) and 87.4% recall, significantly outperforming baseline YOLOv8n by 3.7% in AP while maintaining minimal parameter growth (3.34M). Controlled illumination tests confirm enhanced robustness under strong and warm lighting conditions, with performance gains of 1.5% and 0.7% in AP, respectively. This high-precision framework enables real-time recognition of standing, prone lying, lateral lying, and feeding behaviors in commercial piggeries, supporting early health anomaly detection through non-invasive monitoring.

## Simple Summary

The behavior of pigs can indirectly reflect their health and welfare. Continuous monitoring of these behaviors helps farm managers detect diseases early and safeguard animal well-being. However, traditional manual observation methods are inefficient and may cause stress to the animals. To address this issue, we developed a new automated behavior recognition system based on artificial intelligence. This system accurately identifies four key pig behaviors—standing, prone lying, lateral lying, and feeding—from real-time video surveillance. Experimental results show that the model not only achieves high recognition accuracy but also adapts well to common lighting variations in pig pens, such as strong and warm light. This system provides a non-contact and efficient monitoring tool for farm managers, supporting the transition toward intelligent and welfare-oriented precision livestock farming.

## 1. Introduction

Pigs constitute the primary global source of meat products, and increasing societal development has heightened focus on pork quality [1]. Animal behavior serves as a critical indicator of health status and environmental welfare, with abnormal behaviors often signaling disease onset. Consequently, behavioral monitoring provides early health warnings and enables preventive veterinary interventions [2], establishing its fundamental significance in livestock management [3].

Automated behavior recognition is becoming increasingly significant for enhancing efficiency in smart livestock management [4]. In contrast, traditional methods based on manual observation pose notable limitations due to inherent subjectivity, high labor costs, and the inability to provide continuous 24-h monitoring [5]. Although radio frequency identification (RFID)-based systems, such as the study by Marcon et al. [6], can detect feeding behavior via ear tag signals, they are associated with high costs and may induce animal stress. In contrast, deep learning technology enables non-contact, high-precision behavior recognition, effectively overcoming these limitations [7]. For instance, Alameer et al. [8] employed a GoogLeNet network to achieve non-nutritive visiting (NNV) detection with an accuracy of 99.4% without the need for individual tracking. Li et al. [9] improved the YOLOv5-KCB model, increasing the recognition accuracy of pig heads, necks, and faces to 98.4% and 95.1%, respectively. Zhang et al. [10] proposed an LSR-YOLO model for accurate sheep face recognition, achieving a mAP@50 of 97.8%. Hu et al. [11] introduced a spatio-temporal feature-integrated Transformer model, PB-STR, for simultaneously recognizing seven types of pig behaviors in intensive farming environments. Zhao et al. [12] proposed an improved lightweight model based on YOLOv11, termed ECA–YOLO. This study constructed a high-quality dataset based on hormone detection labels, validating the feasibility of ocular features in estrus recognition, with a mAP@50 reaching 93.2%, outperforming most existing methods. However, the model’s performance degrades to some extent under extreme lighting conditions (e.g., strong light or nighttime) or when significant changes in animal posture occur. Additionally, due to limited breed diversity in the training dataset, its generalization capability remains to be improved. Moreover, the model’s large parameter size is not conducive to on-site deployment. Although the aforementioned deep learning methods address the limitations of manual observation and the instability of traditional image processing techniques [13], they still face several challenges: difficulty in detecting small and occluded targets in complex backgrounds, limited generalization across varying lighting and breeding environment configurations, and high computational costs hindering on-site deployment.

YOLOv8, a state-of-the-art iteration released by Ultralytics in 2023, achieves an optimal balance between accuracy and inference speed. Building upon its predecessors, it incorporates novel optimizations that enhance its versatility for a broad spectrum of object detection applications [14].

Given its favorable balance, we select the lightweight YOLOv8n version as our baseline model to address the challenges of on-site deployment. To further enhance its performance for our specific task, this study proposes a novel multi-scene lightweight framework for recognizing four key pig behaviors (standing, lying on the belly, lying on the side, and feeding). The main contributions of this research include (1) integrating the SPD-Conv module to preserve spatial details during downsampling, thereby enhancing small target detection performance in low-resolution images [15]; (2) incorporating the LSKBlock attention mechanism to improve cross-layer feature fusion; and (3) designing a dedicated small target detection head to achieve multi-granularity behavior recognition.

## 2. Materials and Methods

### 2.1. Dataset Construction

**Figure 1:**
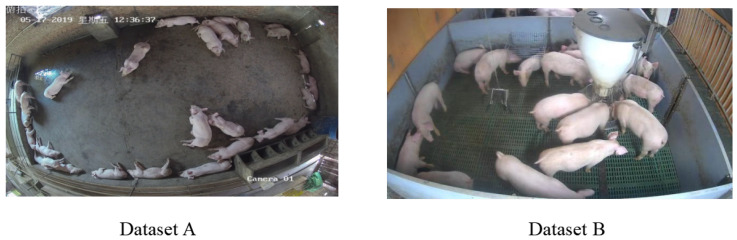
Examples of datasets.

This study utilizes two publicly available datasets, with representative examples illustrated in Table 1. The camera configurations and acquisition parameters are detailed in Table 1. The composite dataset encompasses four distinct scenarios containing pigs exhibiting varying target sizes (large, medium, small), as systematically categorized in Table 1 and Figure 1.

Image acquisition spanned three daily intervals: 10:00–13:00, 16:00–18:00, and supplementary evening periods. This temporal distribution captures behavioral variations between feeding and non-feeding phases. The dataset further incorporates diverse housing configurations—including ground pens and elevated cages—with varied feeding equipment, enhancing image heterogeneity to improve model generalization, robustness, and practical applicability.

A time-conscious data collection strategy is fundamental to our goal of reliably identifying the four target behaviors: standing, feeding, prone lying, and lateral lying. These behaviors are not randomly distributed but follow predictable diurnal rhythms. Concentrating monitoring on peak activity windows (e.g., 09:00–11:00 and 15:00–17:00) ensures high probability of capturing standing and feeding, while also sampling resting postures (prone and lateral lying) within the same periods and across the day [16,17]. This approach ensures that our model is trained on, and evaluated against, a behaviorally complete dataset, thereby increasing the robustness of anomaly detection, such as identifying lethargy (excessive lying) during normally active times [18].

**Figure 2:**
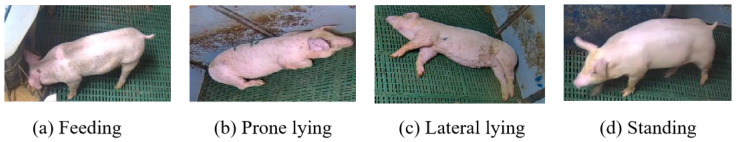
Example diagram of each behavior.

We therefore focus on these four behaviorally validated health indicators (feeding, prone lying, lateral lying, standing) [19]. Classification criteria and representative examples are detailed in Table 2 and Figure 2. All 1820 images were manually annotated using LabelImg [20] with bounding boxes stored in TXT format.

### 2.2. Data Augmentation

**Figure 3:**
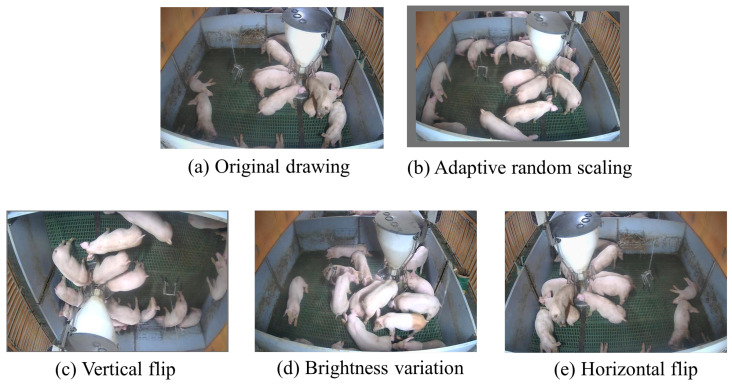
Examples of data augmentation effects: (**a**–**e**).

To enhance model generalization and robustness, and mitigate overfitting [21], we implemented four augmentation techniques: random HSV adjustment, adaptive scaling, horizontal flipping, and vertical flipping (Figure 3).

**Figure 4:**
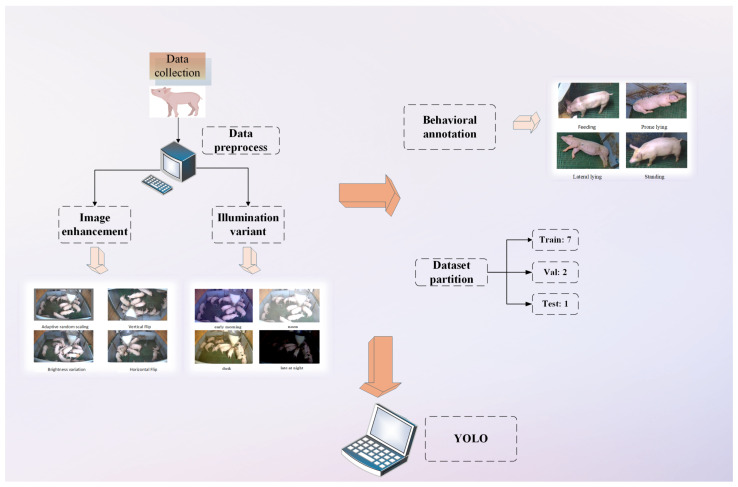
Workflow of the lightweight YOLOv8n-based pig behavior recognition framework.

This pipeline generated 3640 augmented images, expanding the dataset to include 11,258 feeding instances, 11,530 lateral lying occurrences, 13,877 prone lying cases, and 14,599 standing postures. The augmented data was partitioned using stratified random sampling into training (2548 images), validation (728 images), and test sets (364 images) at an 8:2:1 ratio, The overall workflow is shown in Figure 4.

### 2.3. YOLOv8n Network Structure

We evaluated all five variants (YOLOv8n, YOLOv8s, YOLOv8m, YOLOv8l, YOLOv8x) on our pig behavior dataset, with comparative metrics detailed in Table 3.

Although YOLOv8l and YOLOv8x marginally surpassed YOLOv8n in average precision (AP) and mAP@0.5 (by 0.050 and 0.031, respectively), this improvement came at substantial computational cost—the parameter counts increased to 43.6 M and 67.1 M, representing 14.5× and 22.3× expansions relative to YOLOv8n. In contrast, YOLOv8n maintained competitive accuracy (AP = 0.849, mAP@0.5 = 0.896) with only 3.01 M parameters, establishing significant advantages for resource-constrained deployment environments.

**Figure 5:**
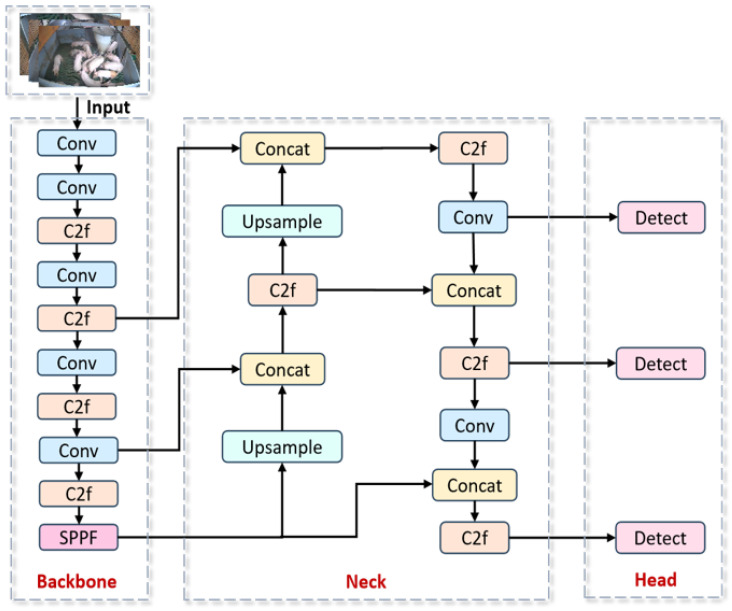
Schematic diagram of the YOLOv8n model architecture.

Furthermore, YOLOv8n’s lightweight architecture confers significant advantages in inference speed [22], satisfying the stringent real-time requirements of practical deployment scenarios. Balancing detection accuracy, computational efficiency, and application constraints [23], we selected YOLOv8n as the foundational framework for this investigation. The architectural configuration is presented in Figure 5.

The YOLOv8n architecture consists of four core components: Input: receives preprocessed pig images; Backbone: performs feature extraction using Conv layers for initial downsampling; Neck: integrates the C2f module for multi-scale feature optimization and SPPF for contextual feature fusion; Head: generates detection outputs through feature pyramid construction.

The processing pipeline initiates with Conv-based downsampling [24], followed by the backbone’s C2f module, which enhances multi-scale representations through concatenated feature optimization. Subsequent feature fusion via the SPPF module enriches contextual information. Higher-resolution feature maps are then recovered through upsampling, enabling hierarchical feature integration where deep semantic features merge with shallow spatial details via concatenation [25]. Finally, detection heads produce 80 × 80, 40 × 40, and 20 × 20 pixel feature maps specialized for small, medium, and large targets, respectively [26].

### 2.4. Improvement of YOLOv8n Pig Daily Behavior Recognition Model

**Figure 6:**
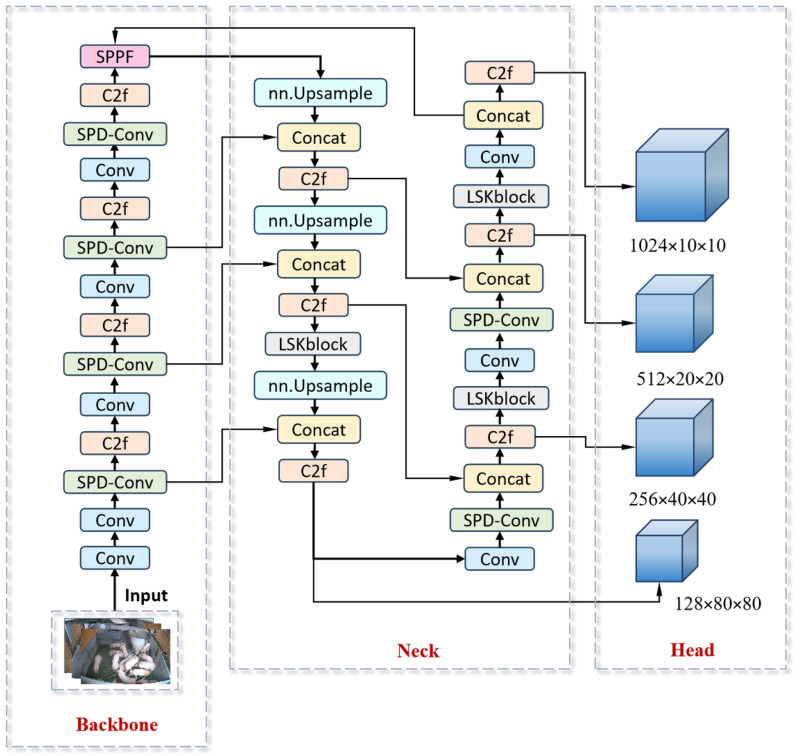
Architecture of the improved YOLOv8n model.

To resolve the persistent challenge of small target loss in pig detection, we integrate the SPD-Conv module, which mitigates information loss during downsampling by preserving local detail features while reducing spatial dimensions. For addressing occlusion and overlapping among pigs, the LSKBlock module [27] is incorporated within the neck section. This component captures long-range dependencies through large-kernel convolutions, dynamically recalibrates feature maps, enhances critical feature propagation pathways, and strengthens spatial perception in complex backgrounds—collectively improving robustness for detecting occluded, overlapping, and small targets [28]. Additionally, a dedicated small-target detection head preserves fine-grained pixel-level details, mitigates information degradation during downsampling, and enhances sensitivity to minute features. These comprehensive architectural modifications are visually summarized in Figure 6.

### 2.5. SPD-Conv Spatial Depth Conversion Convolution

The mixed dataset, reflecting complex pig farming environments, presents challenges including crowded and overlapping pigs, as well as obstructions from facilities like feeding troughs. The dataset also has blurry images or small pig targets in corners. It is necessary to address the issues of low image resolution and neglect of small target tasks during training and testing, as well as information loss caused by traditional downsampling, resulting in poor model training performance [29]. Therefore, in the selection, an SPD-Conv spatial depth conversion convolution module is added after the original Conv convolutional layer [30].

SPD-Conv follows a non-stride convolutional layer from space to depth by introducing multiple convolution kernels with different receptive field sizes into the network, converting image spatial information into depth information and increasing the depth of feature maps without losing information, thereby enhancing the network’s perception ability for targets at different scales. By preserving more spatial and image granularity information when processing low-resolution images and small targets, algorithms can reduce missed detections and improve recognition accuracy [31].

**Figure 7:**
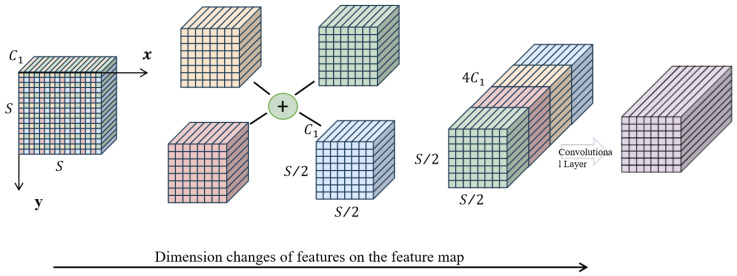
SPD-Conv network structure diagram.

The SPD-Conv module consists of two parts: a space to depth layer and a non-cross row convolution (Conv) layer. The specific structure and principle of SPD-Conv when scale = 2 are shown in Figure 7.

Firstly, a feature map with a size of S × S × C1 is input and downsampled with a step size of 2 to obtain four subfeature maps containing global spatial information.

$$\begin{array}{cc} & f_{0,0}=X\left[0:S:\mathrm{scale},0:S:\mathrm{scale}\right],\;f_{1,0}=X\left[1:S:\mathrm{scale},0:S:\mathrm{scale}\right],\;\ldots ,\\ & f_{\mathrm{scale}-1,0}=X\left[\mathrm{scale}-1:S:\mathrm{scale},0:S:\mathrm{scale}\right];\\ & f_{0,1}=X\left[0:S:\mathrm{scale},1:S:\mathrm{scale}\right],\;f_{1,1}=\ldots ,\;\ldots ,\\ & f_{\mathrm{scale}-1,1}=X\left[\mathrm{scale}-1:S:\mathrm{scale},1:S:\mathrm{scale}\right];\\ & f_{0,\mathrm{scale}-1}=X\left[0:S:\mathrm{scale},\mathrm{scale}-1:S:\mathrm{scale}\right],\;f_{1,\mathrm{scale}-1}=\ldots ,\;\ldots ,\\ & f_{\mathrm{scale}-1,\mathrm{scale}-1}=X\left[\mathrm{scale}-1:S:\mathrm{scale},\mathrm{scale}-1:S:\mathrm{scale}\right]; \end{array}$$

Next, the four subfeature maps are merged along the channel dimension, with the spatial dimension becoming half of the original and the channel dimension becoming four times the original. This can convert the spatial dimension of the input image into a depth dimension, avoiding the problem of information loss caused by traditional cross row convolution and pooling operations. Finally, a non-cross row convolution with a stride of 1 is used to obtain a feature map with a size of S/2 × S/2 × C2.

Non-cross row convolution extracts image features by maintaining the original feature map size and reducing the feature map channels, capturing key information about pig behavior in low-resolution images, achieving fusion from a compressed spatial dimension to channel dimension, improving algorithm performance and reducing information loss while reducing model parameters, computational complexity, and memory usage.

### 2.6. LSKBlock Large Core Space Attention Module

For overlapping occluded pigs and small target pigs in the dataset, using large kernel convolution can cover a larger area and enhance the model’s understanding of the overall structure of the target. And by focusing on the area where the pigs are located through spatial attention, the interference of the background on the model is reduced [32]. Therefore, LSKBlocks were inserted after detection heads at different scales to optimize the feature expression at each level of the model [33].

**Figure 8:**
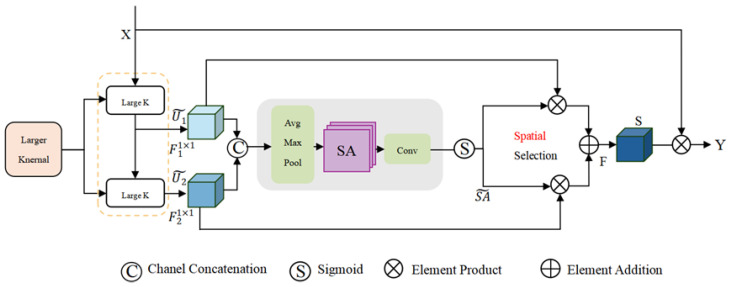
LSK attention mechanism module.

LSKBlock (Large Kernel Spatial Attention Block) is a neural network module that combines a large convolutional kernel with spatial attention mechanism, aiming to enhance the model’s perception ability of key regions, especially suitable for improving the performance of small object detection and complex scenes. The specific structural principle is shown in Figure 8.

The intermediate input feature $X\in R^{C\times H\times W}$, LSK will derive an attention map, and the entire attention process can be summarized as

Y=S(X)⊗X

Here, ⨂ represents element wise multiplication. The specific implementation steps of the LSK module are as follows: Firstly, N deep convolutional layers of different scales are applied to the input feature X to obtain the output of each layer $U_{i}$. This can expand the receptive field and enhance the network’s ability to focus on relevant spatial contextual regions when detecting targets. Then, $U_{i}$ is sent to the standard convolutional layer for further processing and connected together to form $\tilde{U}$:

$$U_{0}=X,\;U_{i+1}=F_{i}^{\mathrm{dw}}\left(U_{i}\right)$$

$$\tilde{U}=\left[F_{1}^{1\times 1}\left(U_{1}\right);\ldots ;F_{i}^{1\times 1}\left(U_{i}\right)\right]$$

Here, $\{F_{1}^{i}\left\{1\times 1\right\},F_{1}^{i}\left\{\mathrm{mdw}\right\}$ represents depthwise separable convolutions with different large convolution kernels, and represents standard convolutions with convolution kernel sizes of 1×1. For widetime[0] applying average pooling and max pooling operations along the channel axis, two different spatial context descriptors can be obtained—$F_{\mathrm{avg}}\in R^{1\times H\times W}$ and $F_{\max}\in R^{1\times H\times W}$—which, respectively, describe the average pooling features and max pooling features across channels. In order to effectively extract spatial relationships $F_{\mathrm{avg}}^{s}$ and achieve information exchange between different spatial context descriptors, the AND $F_{\max}^{s}$ connection is sent into the convolutional layer to obtain N spatial attention maps. For all spatial attention maps, we apply the sigmoid activation function to obtain independent spatial attention masks ${\mathrm{SA}}_{1}\left\{1\times 1\right\}$, then, with $U_{i}$, perform dot product operations and superimpose them together, and send them to the standard convolutional layer for fusion to generate attention maps $R^{C\times H\times W}$:

$$SA_{\left(1\ldots N\right)}=\sigma \left(F^{2\to N}\left[F_{\mathrm{avg}}^{S},F_{\max}^{S}\right]\right)$$

$$S=F^{1\times 1}\left(\sum_{i=1}^{N}\left(SA_{i}\;\cdot \;U_{i}\right)\right)$$

Here, sigma represents the Sigmoid function, and $F^{2\to N}$ represents the standard convolution with input channel 2 and output channel N. The LSK attention mechanism enables the model to focus on important features and ignore unimportant features (highlighting important features and suppressing unimportant features). This is also the key role of the attention mechanism in enhancing network learning ability.

### 2.7. Multi-Scale Feature Detection

**Figure 9:**
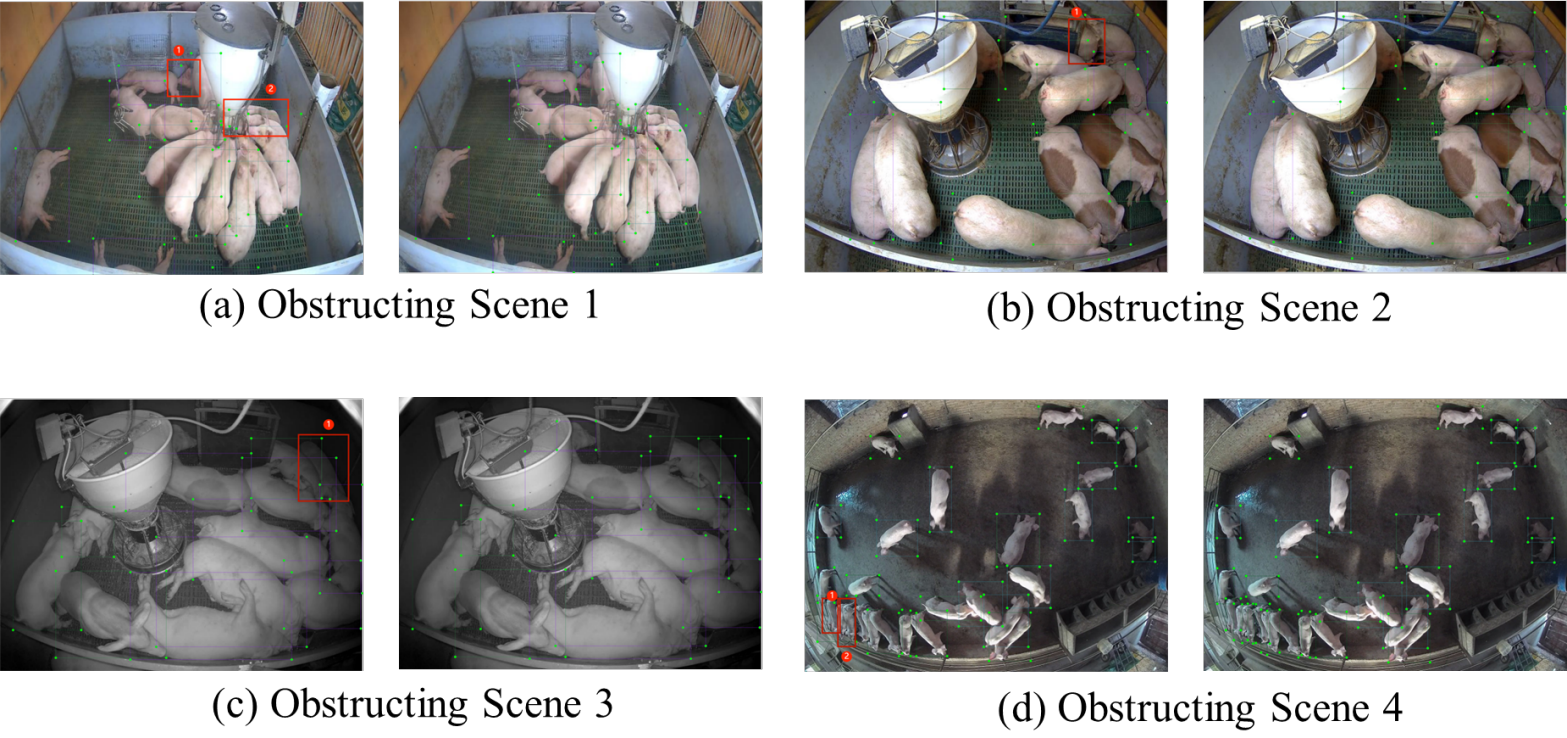
Performance comparison of models before and after adding detection layers in different pigsties.

The baseline YOLOv8n architecture employs three detection heads operating at 80 × 80, 40 × 40, and 20 × 20 pixel scales to detect small, medium, and large targets, respectively, from 640 × 640 input images [34]. However, progressive resolution reduction through deeper convolutional layers causes geometric information degradation, particularly detrimental for small targets like pigs where critical spatial features may be entirely lost [35]. To address this limitation, we introduce an additional small-target detection head that processes higher-resolution features (Figure 9). This enhancement recuperates high-resolution features through successive upsampling, followed by cross-layer concatenation fusing shallow backbone features with neck upsampling outputs. Subsequent channel compression and feature optimization enable the generation of a dedicated 10 × 10 detection head. The resulting four-scale feature pyramid (80 × 80, 40 × 40, 20 × 20, 10 × 10) [36], combined with LSKBlock’s attention mechanism, dynamically focuses on small-target regions while suppressing background interference.

### 2.8. Experimental Parameter Settings

All computational experiments were conducted on a Windows 10 system with 64-bit architecture using Python-based frameworks. The hardware configuration comprised an Intel Core i5-8300H CPU (2.30 GHz) (Intel Corporation, Santa Clara, CA, USA) paired with an NVIDIA GeForce RTX 4090 GPU (8GB VRAM) (NVIDIA Corporation, Santa Clara, CA, USA). The software environments included a Conda-managed Python 3.10.16 installation with PyTorch 2.0.1 and CUDA 11.8. The detailed training hyperparameters are provided in Table 4.

The evaluation metrics comprise precision (P), measuring the correct target recognition proportion; recall (R), indicating the detection completeness; and mean average precision (mAP), quantifying the overall categorical accuracy [37]. The model’s performance was objectively assessed through parameter efficiency and these established metrics across four critical porcine behaviors: standing, feeding, prone lying, and lateral lying.

## 3. Results

### 3.1. Ablation Test

Ablation studies validated the contributions of the enhanced model using the original YOLOv8n as baseline. The SPD-Conv module, LSKBlock attention mechanism, and small-target detection head were incrementally integrated into the baseline architecture to assess performance improvements [38], with the comprehensive results documented in Table 5.

Integrating the SPD-Conv module yielded significant metric improvements: precision (P) increased by 2.2%; mean average precision, at 0.5 IoU (mAP50), by 1.8%; and recall (R), by 3.6%, despite a marginal parameter increment. Subsequent incorporation of the LSKBlock attention mechanism further elevated P by 0.016 percentage points and mAP50 by 0.005 absolute points, though the parameter complexity doubled and R experienced a slight reduction. Final integration of the small-target detection head produced a marginal P decrease (0.001%) but improved the mAP@50 and R by 0.005 and 0.01 percentage points, respectively, again doubling the parameter count. Collectively, the enhanced model demonstrated substantial gains across all metrics versus the YOLOv8n baseline, with parameter increases remaining within practical constraints for lightweight deployment.

### 3.2. Analysis of Recognition Results of Different Models

Comparative analysis against prominent YOLO variants demonstrates the superiority of our enhanced model. As quantified in Table 6, the proposed architecture achieves significantly higher mean average precision (mAP@0.5 = 0.924) and recall (R = 0.874) than the computationally comparable YOLOv5n (mAP@0.5 = 0.862, R = 0.8), with only a 10% parameter increase. While YOLOv3 exhibits marginally superior performance in certain metrics, this advantage is offset by its substantially larger parameter footprint (96% greater than our model), rendering it impractical for lightweight deployment. Crucially, our solution outperforms YOLOv5n by 6.2 percentage points in mAP@0.5 while maintaining favorable computational characteristics.

Further validation through porcine behavior recognition reveals our model’s exceptional accuracy for clinically significant behaviors: feeding (AP = 0.912) and prone lying (AP = 0.932)—critical indicators of health status where performance degradation often signals early pathology [39]. Though lateral behavior detection trails YOLOv3 by a marginal 0.3% (0.904 vs. 0.905), our implementation achieves this with a 96% parameter reduction, establishing superior deployment efficiency for resource-constrained environments as shown in Table 7.

**Figure 10:**
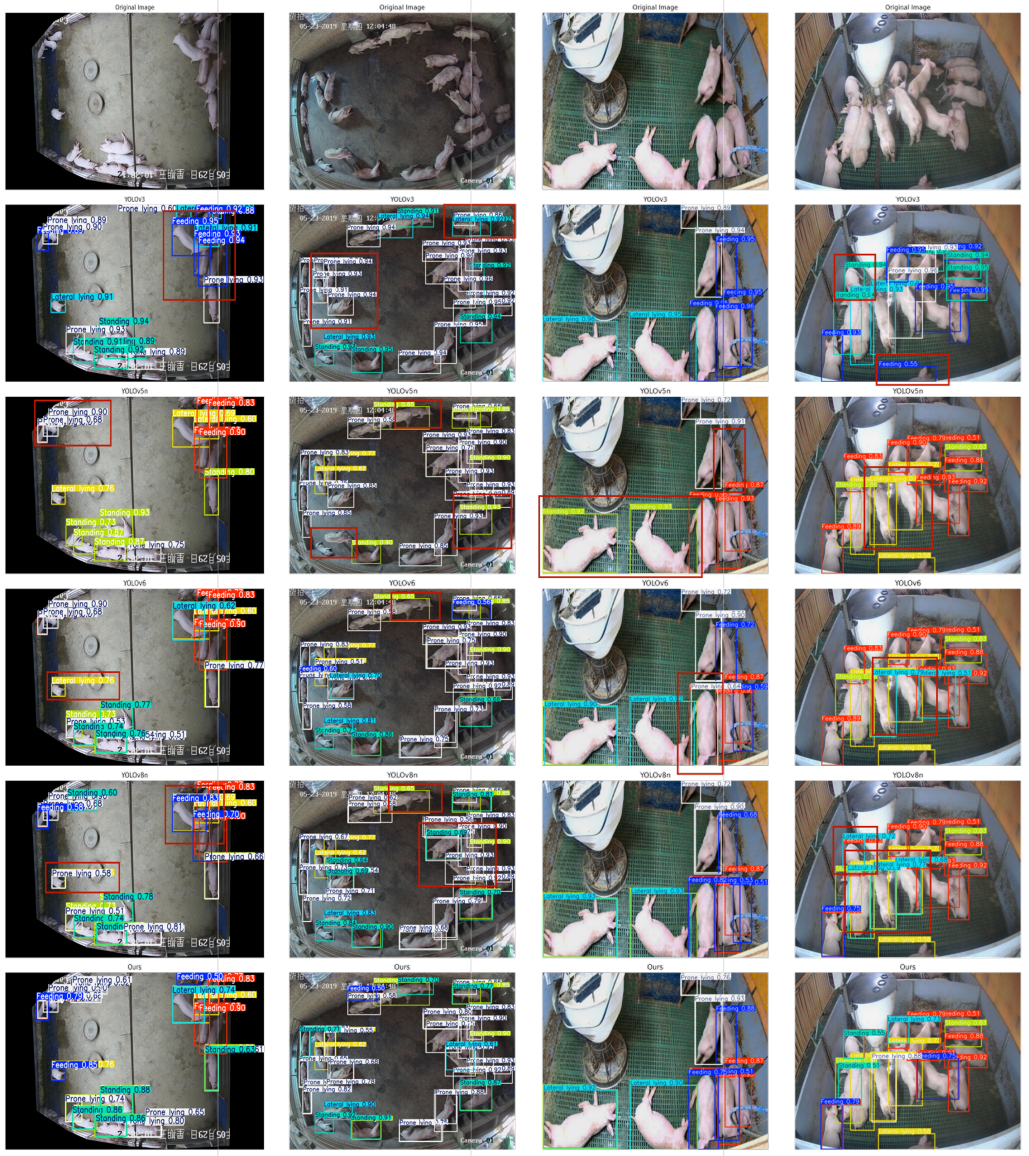
Comparison of detection effects of different models (red boxes indicate false positives or false negatives).

Figure 10 presents a comparative visualization of four representative pigsty scenes processed through distinct detection models: original imagery followed by YOLOv3, YOLOv5n, YOLOv6, and YOLOv8n outputs. Thickened red bounding boxes highlight false positives and missed detections across all models, revealing non-zero false positive and false negative rates in each implementation.

### 3.3. Multi-Scenario Robustness Verification of the Model

#### 3.3.1. Comparison of Model Performance Under Different Lighting Conditions

**Figure 11:**
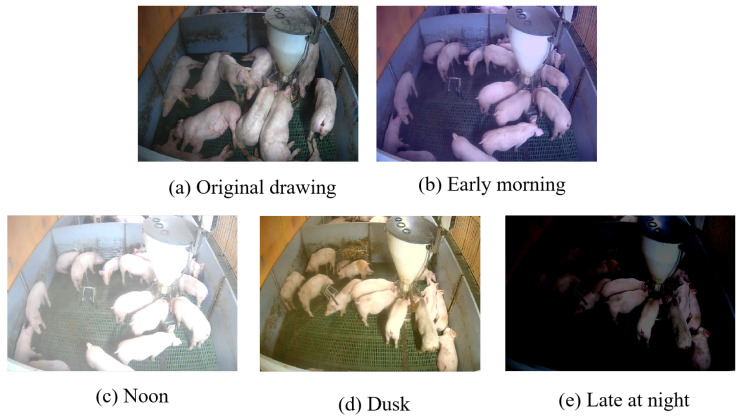
Simulation diagram of light rays at different time periods.

To assess model robustness across varying illumination conditions, 50% of the mixed dataset images were randomly selected for synthetic lighting transformation. We generated photorealistic variants simulating four diurnal phases—early morning, noon, dusk, and late night—through automated batch processing. Representative examples of these illumination scenarios are presented in Figure 11.

As presented in Table 8, the improved model demonstrates consistent performance enhancements over the original model under strong light, warm light, and cold light conditions. This improvement validates the LSKBlock’s capability for long-range contextual modeling and the SPD-Conv module’s effectiveness in preserving spatial detail. However, performance degradation was observed in low-light scenarios (ambient illumination < 10 lux). This decline is likely attributable to several factors: a reduction in CMOS sensor quantum efficiency at these illumination levels, resulting in a signal-to-noise ratio (SNR) below 4 dB [40]; the tendency of LSKBlock’s large kernel selection mechanism to span multiple noise regions under low light; and the submersion of key features—such as the contour curvature characteristics of small target pigs and recumbent postures—by noise. These features, preserved by SPD-Conv under low light, effectively form structural noise. Furthermore, thermal signatures from aggregated pigs and the inherent difficulty of capturing pigs with black skin in dark conditions exacerbate these challenges.

To quantitatively assess the models’ focus on pig behavior across lighting conditions, we visualize the feature attention distributions of both the original and improved models using the Grad-CAM method. This analysis employs images from four representative pigsty scenes: cool_tone, strong_light, warm_tone, and low_light. The resulting attention heatmaps highlight the high-level features learned by each model.

**Figure 12:**
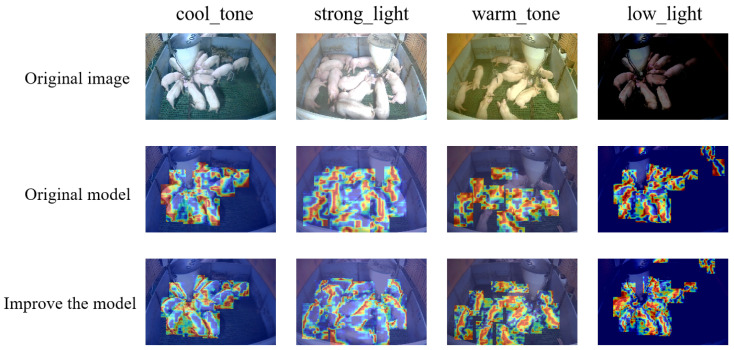
Thermal map comparison between the original model and the improved model under different lighting conditions.

As illustrated in Figure 12, the original images depict the actual distribution of pig herds across varying lighting conditions. The corresponding attention heatmaps from the original model reveal fragmented and incomplete focus regions. Under low-light conditions, the original model exhibits insufficient attention coverage for pigs in darker areas. In brightly lit scenes, feature overexposure causes confusion within the heatmap’s focus.

The enhanced model, incorporating the SPD-Conv module, LSKBlock module, and optimizations for small object detection, demonstrates significant improvements in attention mapping. In cool-tone scenes, the generated heatmaps now accurately encompass the full contours of pigs while providing clearer distinction between standing and lying postures. Crucially, under low-light conditions, the improved model effectively captures porcine features in dark regions. These visualizations validate the enhanced strategy’s improved lighting robustness and superior feature attention integrity.

#### 3.3.2. The Differential Effect of Light on the Recognition of Different Behaviors in Pigs

To investigate the specific effects of lighting conditions on the four target behaviors, we present detailed quantitative results in Table 9. The data reveal consistent performance improvements across all lighting conditions except low light, with particularly notable enhancement in front-lying detection under strong illumination. This improvement likely stems from the enhanced contour curvature features of pigs under high light levels, facilitating model recognition. However, eating behavior detection declined across all lighting scenarios. Under low-light conditions, motion blur increases signal-to-noise ratio loss in the snout region, impeding recognition. Strong lighting induces specular reflections from metal troughs [41], interfering with model performance. Warm lighting conditions introduce confusion in the 590 nm yellow light band due to spectral similarity with pig skin tones.

## 4. Discussion

The proposed lightweight framework, building upon YOLOv8n, achieved a competitive mAP@50 of 92.4% for multi-behavior recognition, with a particularly high accuracy of 94.9% in identifying standing posture. This performance demonstrates the effectiveness of our architectural modifications in addressing key challenges in complex farming environments.

The integration of the SPD-Conv module was essential for preserving fine-grained spatial details. Its spatial-to-depth transformation effectively reduced the limb edge blurring typically associated with standard downsampling. This improvement is crucial for distinguishing subtle posture differences in pigs, such as between prone and lateral lying. The model’s strong performance in detecting small-target behaviors provides quantitative support for this enhancement.

Furthermore, the introduction of the LSKBlock attention mechanism improved feature discrimination across different scales. Its adaptive receptive field capability helped suppress background interference and enhance the focus on distinctive animal contours, leading to robust recognition under varying scene complexities. The high recognition accuracy across all four behaviors indicates that the combined use of SPD-Conv and LSKBlock successfully balanced detailed feature preservation and contextual feature extraction.

When compared with existing literature, the performance of our proposed framework is competitive with, and in some aspects surpasses, that of more complex architectures reported in prior studies. For example, the ECA–YOLO model by Zhao et al. achieved a mAP@50 of 93.2% for sow estrus detection, but their model faced challenges regarding computational complexity and deployment feasibility. Similarly, although the PB-STR Transformer model by Hu et al. can recognize seven behaviors simultaneously, its high computational demand may limit its practical application in resource-constrained environments. In contrast, our model achieves a compelling balance between accuracy and efficiency, obtaining a competitive mAP@50 of 92.4% while being based on a much lighter architecture, thereby showing greater potential for on-site deployment.

While the results are satisfactory, certain limitations of the model cannot be denied. As mentioned earlier, under extreme lighting conditions or severe occlusion, the model’s performance may degrade. This indicates that, although our current feature extraction approach has been improved, it is not yet immune to such disturbances. In addition, the generalization ability of the model across different pig breeds requires further validation with more extensive datasets.

In summary, this study demonstrates that a strategically modified lightweight architecture can achieve high-precision pig behavior recognition. The successful integration of SPD-Conv, LSKBlock, and the small-object detection head provides a valuable reference for detecting pigs in agricultural settings. Future work will focus on enhancing the model’s robustness to environmental variations and exploring its real-time deployment on edge computing devices.

The model achieves a recognition accuracy of 91.2% for pig feeding behavior, enabling real-time monitoring of appetite variations and systematic logging of feeding patterns. When feeding duration decreases by more than 15%, the system alerts managers to potential disease risks. This behavioral precision feeding approach simultaneously optimizes feed utilization. Furthermore, empirical evidence indicates that the ratio of lateral to prone lying positions reflects environmental comfort levels within the pigsty. During elevated temperatures, pigs exhibit a preference for the prone position to facilitate abdominal heat dissipation, while under cooler conditions, they favor lateral recumbency to minimize body surface contact with the ground [42]. Consequently, the model’s continuous detection and statistical analysis of lateral and prone posture proportions provide valuable data for informing environmental control decisions within the pigsty.

The model’s varying performance under different lighting conditions offers important insights into its strengths and limitations. While it performs very well under warm and strong light, where the LSKBlock and SPD-Conv work effectively together, its performance drops by about 1.2% in low-light situations. This highlights a significant challenge for real-world use. We believe that this decline is not only due to limited data but is also related to the model’s design. In low light, the large kernels in the LSKBlock may combine noisy areas, creating false features. At the same time, the SPD-Conv module, which normally preserves important details, can also amplify sensor noise and heat signatures from groups of animals. Additionally, the consistently lower detection rate for feeding behavior across all lighting conditions suggests that this activity may involve finer details that are not fully captured by our current features. The higher missed detection rate for darker-skinned pigs in low light further indicates a need for more diverse datasets to improve fairness and generalization. Therefore, future work should focus not only on adding more data but also on designing better models that can adapt to different lighting and reduce the impact of noise.

Under strong light, the contours of standing pigs become more distinct, leading to a clear improvement in detection performance. In contrast, feeding behavior is more susceptible to factors such as reflections, motion blur, loss of mouth detail, and glare from metal troughs. Therefore, it is recommended that future studies employ local enhancement techniques or multi-modal fusion (e.g., with infrared imaging) to address these challenges.

The limitations identified in this study, particularly under low-light conditions and with complex behavioral sequences, point the way for future research. To overcome these constraints, subsequent work will focus on three main directions. First, building on the real-time capability of our lightweight framework, we will expand the range of detectable behaviors to cover more welfare indicators. Second, and most importantly, the performance drop in low light strongly supports the integration of infrared imaging. This will help create a detection module that works regardless of lighting, directly addressing a key weakness found in our experiments. Finally, to go beyond static posture recognition, we plan to incorporate Transformer-based models to analyze behavioral sequences over time, such as feeding and excretion. This step from single-frame detection to sequence analysis is essential for progressing to predictive anomaly detection and smarter management systems.

Our model can accurately identify pig postures such as standing and lying, making automated behavior monitoring more practical. For example, it reliably detects standing behavior with 94.9% accuracy, which can help managers identify health issues early and intervene promptly. If a pig’s standing pattern changes, the system can alert managers to potential problems such as lameness. The model also distinguishes between lateral and prone lying postures, providing an objective way to assess animal comfort and welfare. Although full integration with veterinary knowledge for computer-aided diagnosis remains a future goal, our model offers a robust tool for collecting high-quality, long-term behavioral data. This data is essential for linking specific behavioral changes to diseases. Overall, this study not only provides an effective recognition model but also supports the transition toward data-driven management strategies that can improve both animal welfare and farm profitability.

## 5. Conclusions

This study presents a multi-scene pig behavior recognition model based on YOLOv8n. The model integrates an SPD-Conv module to preserve details in low-resolution images, introduces an LSKBlock mechanism to enhance robustness in complex environments, and adds a new small-object detection head. Ablation studies and comparisons confirm that the optimized model remains lightweight (3.34 M parameters) while achieving a 0.901 mAP@50 and a 0.907 recall rate, enabling accurate pig behavior recognition. This work provides an effective tool for non-contact monitoring in modern intelligent pig farming.

## Figures and Tables

**Table 1:** Example images from (A) Dataset A and (B) Dataset B.

Name	Source	Main Shooting Location and Angle	Number of Scenes	Target Size of Pigs
A	Public dataset	The side of the pigsty is 3 m high and photographed from a downward angle	2	Medium and small targets
B	Public dataset	The side of the pigsty is 2 m high and photographed from a downward angle	2	Medium and large goals

**Table 2:** Examples of the four classified pig behaviors: feeding, prone lying, lateral lying, and standing.

Behavior	Characteristic
Feeding	Standing in front of the trough, inserting the head into the trough.
Prone lying	Lying flat with limbs extended, and head and forelimbs usually straight forward, lying on the ground with the abdomen tightly attached to the ground.
Lateral lying	Lying on one side, with limbs extended, and head and neck straight in line with the body.
Standing	In an upright position, with the limbs supporting the body, while the head and neck maintain an upright or slightly forward-leaning posture.

**Table 3:** Comparison of detection data for YOLOv8 models in different versions.

Models	P (%)	mAP50 (%)	R (%)	Parameters
yolov8n	0.849	0.896	0.829	$3.01\times 10^{6}$
yolov8s	0.892	0.931	0.891	$1.22\times 10^{7}$
yolov8m	0.905	0.932	0.901	$2.58\times 10^{7}$
yolov8l	0.899	0.934	0.905	$4.36\times 10^{7}$
yolov8x	0.902	0.927	0.894	$6.81\times 10^{7}$

**Table 4:** Training parameter settings.

Parameter	Value
Epochs	200
Batch size	16
Learning rate (lr0)	0.001
Optimizer	Adam
Weight decay	0.0005

**Table 5:** Results of ablation experiments.(“-” indicates that the corresponding module is not added, while “✓” indicates that the corresponding module is added).

Models	SPD	LSK	Small Head	P (%)	mAP50 (%)	R (%)	mAP50–95 (%)	Params
YOLOv8n	-	-	-	0.849	0.896	0.829	0.732	3.01 M
+SPD	✓	-	-	0.871	0.914	0.865	0.776	3.45 M
+SPD+LSK	✓	✓	-	0.887	0.919	0.864	0.788	6.61 M
Full model	✓	✓	✓	0.886	0.924	0.874	0.785	3.34 M

**Table 6:** Comparison of model performance metrics.

Model	P (%)	R (%)	mAP50 (%)	mAP50–95 (%)	Parameters
yolov3	0.903	0.866	0.924	0.821	$1.04\times 10^{8}$
yolov5n	0.860	0.8	0.862	0.688	$1.76\times 10^{6}$
yolov6	0.820	0.812	0.881	0.705	$4.23\times 10^{6}$
yolov8n	0.849	0.829	0.896	0.732	$3.01\times 10^{6}$
Ours	0.886	0.874	0.924	0.785	$3.34\times 10^{6}$

**Table 7:** Comparison of average accuracy of pig behavior recognition between different models and improved models.

Behavior	YOLOv3	YOLOv5n	YOLOv6	YOLOv8 (Ours)
Feeding	0.911	0.856	0.867	0.912
Lateral lying	0.905	0.813	0.859	0.904
Prone lying	0.927	0.856	0.882	0.932
Standing	0.951	0.921	0.915	0.949

**Table 8:** Model performance comparison under different lighting conditions.

Lighting Conditions	Original Model mAP50%	Improved Model mAP50%	Increase Amplitude	Accuracy Improvement	Recall Rate Increase
Standard lighting	0.896	0.924	+2.8%	+3.7%	+4.5%
Low light	0.606	0.594	−1.2%	−0.4%	−1.0%
Strong light	0.876	0.887	+1.1%	+1.5%	+2.0%
Warm tone	0.896	0.902	+0.6%	+0.7%	+2.2%
Cool tone	0.894	0.899	+0.5%	+0.6%	+2.2%

**Table 9:** Data amplitude changes for different behaviors under different lighting conditions.

Behavioral Categories	Increase in Low Light Intensity	Increase Amplitude of Strong Light Exposure	Warm Lighting Increase Amplitude	Increase in Cold Light Intensity
Standing	−1.1%	+1.1%	+1%	+0.8%
Feeding	−1.4%	−0.4%	−1.7%	−0.1%
Prone lying	−0.9%	+2.2%	+2%	+1.1%
Lateral lying	−1.3%	+1.7%	+1%	+0.7%

## Data Availability

The datasets utilized in this study are publicly available and sourced from the following repositories: **Pig Behavior Dataset:** Available on RoboFlow Universe at https://universe.roboflow.com/swine-tktu8/pig-behavior-8xbgn/dataset/1 (accessed on 28 February 2025); **Pig-Check Challenge Dataset:** Provided by iFLYTEK Co., Ltd. for the “Pig-Check Challenge” (2021). Available at https://challenge.xfyun.cn/topic/info?type=pig-check&ch=ds22-dw-kol (accessed on 28 February 2025). [Original citation: iFLYTEK Co., Ltd. Pig-Check Challenge [EB/OL] (2021-06-21)]. https://challenge.xfyun.cn/topic/info?type=pig-check (accessed on 27 February 2025)].
